# Prevalence of Major Depressive Disorder and Correlates of Thoughts of Death, Suicidal Behaviour, and Death by Suicide in the Geriatric Population—A General Review of Literature

**DOI:** 10.3390/bs11110142

**Published:** 2021-10-21

**Authors:** Gloria Obuobi-Donkor, Nnamdi Nkire, Vincent I. O. Agyapong

**Affiliations:** 1 Department of Psychiatry, University of Alberta, Edmonton, AB T6G 2B7, Canada; Nnamdi.Nkire@albertahealthservices.ca (N.N.); vincent.agyapong@nshealth.ca (V.I.O.A.); 2 Department of Psychiatry, Dalhousie University, Halifax, NS B3H 2E2, Canada

**Keywords:** suicide, major depressive disorder, elderly, prevalence, correlates

## Abstract

**Background:** There has been an increase in deaths by suicide in old age in the last decade. Depression and suicide in the elderly, 60 years and above, is a major global public health concern. Determining the prevalence of depression, and correlates of death by suicide in the geriatric population, is an important first step toward addressing this public health concern. This literature review aims to determine the prevalence of major depressive disorders and the correlates of death by suicide in the geriatric population. **Methods:** This general review of the literature was performed using relevant search terms to determine both the prevalence of depression and the correlates of death by suicide among the geriatric population. Databases such as MEDLINE, PsycINFO, CINAHL, and PubMed were searched. Relevant and current articles were extracted, reviewed, and analyzed. The elderly population was defined as individuals 60 years and above. Only full texts articles in English were reviewed. **Findings:** The prevalence estimates of major depressive disorder in the elderly ranged from 5.37 to 56%. Adults aged 60 years and older have a high risk of depression that exposes them to suicide. Moreover, elderly women are more likely to experience depression than elderly men, but successful suicide is more common in men. Depression and other mental health conditions (schizophrenia, anxiety disorders) and perceived stress were found to be predictors of suicide in the elderly. Other predictors included physical illnesses such as malignancies, financial constraints, cuckoldry, and sexual dysfunction, and also social factors like living alone triggers depressive symptoms and increases suicidal risk in the elderly. Hanging was found to be the most common method of death by suicide for both sexes. While elderly women preferred poisoning, elderly men in Western countries preferred firearms. Differences in gender, the aging process and social issues were also contributing factors to methods used for suicide. **Conclusions:** Depression and debilitating physical illnesses were identified as significant contributors to suicide risk in the elderly population, and emphasis should be placed on identifying these factors early and treating them. Recognizing and addressing factors that predict suicide in the elderly will help to improve the mental wellbeing of the elderly.

## 1. Introduction

Old age is a period involving physical and functional disabilities, as well as a reduction of cognitive function, social life, loss of autonomy, and independence [1]. As one advance in age, the quality of life may reduce with a decline in physical and cognitive abilities or infirmities. Chronic diseases and reduced strength lead to a sense of worthlessness and anxiety, ultimately resulting in depression [2]. The United Nations (UN) Population Division projects an increase of the geriatric population (60 years and above) from 800 million representing 11% of the general population, to 2 billion, i.e., 22% of the population by the year 2050 [3]. In 2008, persons 65 years and over constituted 13.5% of the Ontario population; by 2036, that figure is expected to rise to approximately 23.2% [4]. It is estimated that the prevalence of mental health problems ranges from 17 to 30% or higher, depending on the diagnoses included in the analysis [4]. The elderly population in Ghana accounted for 4.7% of the total population in 2010, with the elderly population making up 56% women and 44% of men [5]. 

Globally, suicide rates in men and women have been reported as 25 per 100,000 persons and 6 per 100,000 persons among men and women, respectively, in the general population [6]. For example, a study in China suggests that the prevalence of suicide among Chinese older adults is on the rise compared to the prevalence estimates in Western countries [7]. A suicide mortality rate of 23 per 100,000, or 287,000 deaths, is reported annually in China [7]. A study of suicides among centenarians in 17 countries revealed a suicide rate of 57 per 100,000 men and 6.8 per 100,000 women [6]. In Germany, the suicide rate in those over 90 years was 90.1 per 100,000 for men and 20.9 per 100,000 for women [6].

Globally, approximately every 40 s, a person dies by suicide somewhere in the world [2]. Suicide attempts in elderly people are more fatal than in young adults [8,9]; therefore, more attention is needed among the geriatric population. The elderly population forms a significant proportion of the population, and with the geriatric population inflating, suicides among the elderly pose a serious issue for global societies. Suicide is the conscious act of inflicting injury to oneself with the behavior and intention to die [10,11,12]. Suicide is a serious public health problem for the elderly [13], however, the suicide rate among the elderly has not been as high as that among adolescents since the 1970s. 

The suicide rate among the elderly is still high, particularly in Western countries such as Canada and the United States [14], although other countries may record lower values of suicide in the elderly. For example, Malaysia records 3.0 per 100,000 suicides among the elderly [15]; reasons may be related to religious practices [15]. Suicide, especially among men in Traditional cultures in China, represents failure, which makes deliberate injuries being recorded as accidents to save one from shame [16]. It is estimated that 7000 elderly aged 60 years and above are involved in suicide yearly in the United States [17].

Persons 65 years and older represent 13% of the general population and account for 18% of deaths by suicide in the year 2000 [10]. In the year 2005, it was estimated that 575,000 persons visited the emergency departments in the US on account of self-injury [17]. Suicide accounted for close to 1.5% of all deaths worldwide, bringing it into the top 20 leading causes of death in 2015 [12]. In the 2010 Global Burden of Disease, self-injury was ranked 17th at age 60–64 years [18] among causes of death.

The self-destruction data in elders are very high, with a close-ratio of nearly 2:1 attempted and completed suicides, respectively [19]. The American Association of Suicidology reported that, among the general population, there is one accomplished death in 25 suicide attempts with a 1:4 ratio among the elderly and 1:100–200 attempts among the youth [20]. These statistics are alarming and make it a health concern to the society and the healthcare sector, leading to calls for action to reduce the risk of such acts [4].

Depression seems to be a significant contributor to suicide, with about 800,000 people dying by suicide yearly [21]. According to the Diagnostic and Statistical Manual of Mental Disorders, fifth edition (DSM-V), depressive disorders include “disruptive mood dysregulation disorder, major depressive disorder, dysthymia, premenstrual dysphoric disorder, substance/medication-induced depressive disorder, depressive disorder due to another medical condition, other specified depressive disorder, and unspecified depressive disorder” [22].

Depression is characterized by the presence of sad, empty, or irritable moods [22]. Usually, the first sign of depression among the geriatric population is a lack of interest in usual activity [23]. In defining depression in both the elderly and younger population, depressive symptoms, specific depressive illnesses or disorders, number and type of symptoms are considered [24].

Depression is more common in people over 65 years old than other emotional changes, accompanied by a high mortality rate and pathological comorbidity [25]. Currently, approximately 20% of women and 10% of men in the USA experience major depressive disorder [25]. A study conducted in Athens shows that, of the cohort of 300 elderly individuals studied, 84.3% showed symptoms of depression in the moderate and severe spectrum of the illness [25]. In a study of about 1300 community adults 60 years or older, results from screened depressive symptomatology showed 27% reported depressive symptoms, 19% were diagnosed as suffering mild dysphoria, 4% symptomatic depression, 2% dysthymia, 1.2% a mixed depressive and anxiety syndrome, and 0.8% major depression [26].

Longitudinal analyses of the practice-randomized Prevention of Suicide in Primary Care Elderly: Collaborative Trial (PROSPECT) analyzed 1226 elderly adults. Results showed 29% of patients with major depression and 11% with minor depression wished to die. Meanwhile, 7% with no depression also had suicidal thoughts [27]. Another study that studied persons 65 years or older recorded major depressive disorder prevalence around 1.3–4%, dysthymic disorder 2%, minor depressive disorder 4–13%, and 8–16% of depressive symptoms [28].

The depressed elderly may fail to express their sadness, and they often tend to somatize their complaints [28] which makes diagnosing depression in the elderly challenging [29]. People’s experiences as they age overlap between the vegetative symptoms of depression and that of comorbid physical illness making accurate diagnoses difficult [29] and leading to unnecessary treatment. To fully evaluate depression among the elderly, validated measures, such as the Patient Health Questionnaire 9 (PHQ-9) [30] and Beck’s depression inventory [31] can reflect diagnostic criteria [32]. The Geriatric Depression Scale (GDS) is also one of the best screening tools for depression in the elderly who are healthy/ill or mild to moderately cognitively impaired [33,34]. These tools indicate significant depression and assess for suicidal risk; the scores help provide adequate intervention for suicide. The elderly adults, peculiarly elderly white men, have high suicide rates, hence, the presence of suicidal thoughts should be carefully investigated [35]. Poor recognition of mental disorders and suicidal ideation among the elderly makes it challenging to get an appropriate solution. Determining the prevalence of major depressive disorder and the predictors of suicide in the geriatric population will support the health of the elderly and improve their quality of life. To the best of our knowledge, this is the first review article to examine the prevalence and correlates of depression and suicide in the elderly as well as explore different methods adopted in elderly suicide. Epidemiological studies, whilst useful in this context, do not specifically explore all the different predictors of suicide or all the various methods used in suicide in the geriatric population. Thus, this general literature review was conducted to synthesize the data regarding depression and suicide as far as it related to the elderly population. Specifically, we aim to explore literature related to the prevalence of depression, demographic, social, and clinical correlate of suicide, and the methods commonly adopted in completed or attempted suicides among the geriatric population.

## 2. Methods

### 2.1. Data Sources

A comprehensive and narrative review of studies and research on depression and death by suicide in the elderly was performed. We appraised suicidal attempts and ideation due to the close relationship of these terms in the geriatric population [36]. This general review of the literature was performed using relevant search terms to determine both the prevalence rates and correlates of depression and death by suicide among the geriatric population. Databases such as MEDLINE, PsycINFO, CINAHL, and PubMed were searched. The study included publications from 1990 to 2021 and most relevant database to the study was MEDLINE and PubMed. Relevant and current articles were extracted, reviewed and analyzed. Articles were screened to find a group of articles that focused specifically on suicide in the elderly and depression in the same group. Qualitative and quantitative studies were included in this review.

#### 2.1.1. Inclusion and Exclusion

Studies were considered eligible when the elderly were defined as persons 60 years of age or older in relevant journals. This placed a cut-off for the age definition of the elderly due to the lower numbers of papers for people aged 70 and above. Articles that specified the reason for suicide in the geriatric populations were included in this review. To be included in our review, a study needed to report the correlation of the predictor of suicide or suicidal behavior in the geriatric population. Priority was given to outcomes directly related to suicide and measures of suicidality; secondary priority was given to depression. Only full texts in English were reviewed.

Articles were excluded if they did not focus on suicide in the elderly or if the article focused on suicide and depression in the general population instead of the geriatric population specifically. However, studies that did not specify the predictor of suicidality among the geriatric population were excluded, and articles that did not fall in our age category of elderly were excluded. Furthermore, editorial, opinion and theoretical articles were excluded from the review. Covidence software was used to (https://www.covidence.org/ (accessed on 15 June 2021 @ Edmonton, Canada)) enhanced the screening and data extraction of the data collected.

#### 2.1.2. Search Strategy

The search strategy included a combination of MeSH terms, keywords, and descriptors: (elderly OR aged OR older OR elder OR geriatric OR elderly people OR old people OR senior) AND (predictors OR indicators or factors OR determinants OR risk factors) AND (depression OR depressive disorder OR depressive symptoms OR major depressive disorder OR MDD OR mood disorder) AND (suicide OR suicidality OR suicidal OR suicidal ideation OR suicidal behaviors OR suicidal thoughts OR death) AND (prevalence or incidence OR epidemiology or frequency OR occurrence). The search included all articles indexed as of 15 June 2021. Duplicates and non peer-reviewed articles were removed before the remaining abstracts were reviewed. The data were systematized into;

1. Prevalence of depression in the geriatric population;
2. Prevalence and predictors of thoughts of death by suicide, suicidal behaviors, and death by suicide in geriatric population;
3. Methods of suicide.

## 3. Results

**Figure 1:**
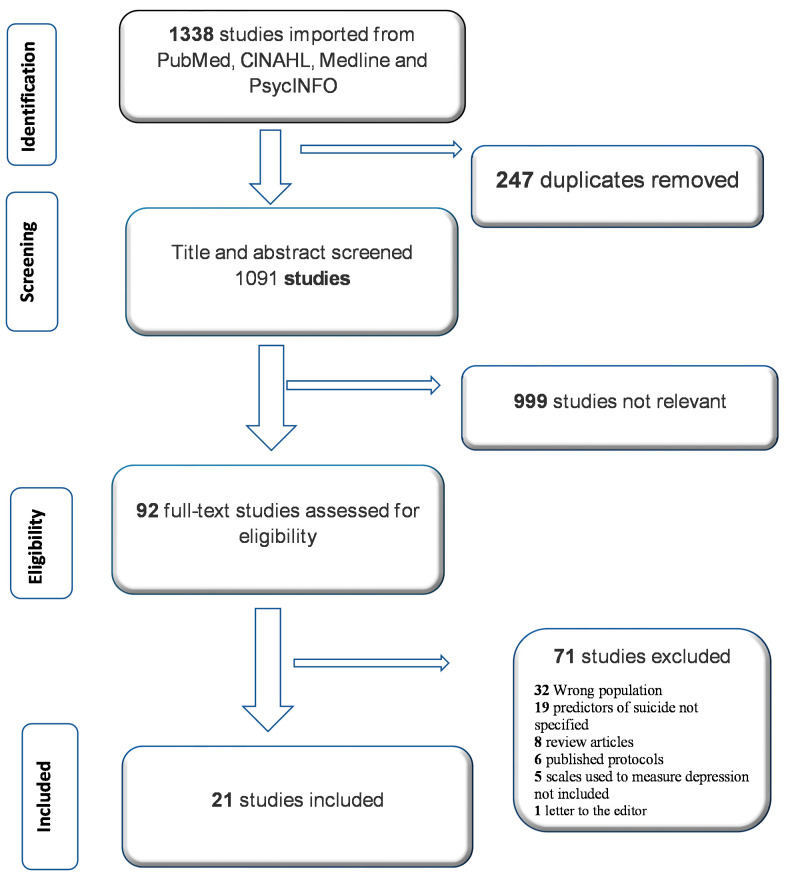
PRISMA flowchart of study selection.

The flowchart in Figure 1 shows that 1340 studies were initially extracted from the identified databases using the Covidence software. Of these, 248 studies were automatically identified as duplicates and removed, leaving 1092 studies screened for eligibility. Further screening was carried out using the inclusion/exclusion criteria, and a full-text review resulted in a final pool of 24 studies that were eligible for inclusion in this literature review.

The twenty-four studies included a total of 306,173 subjects, and the sample size ranged from 40 [5] to 203,668 [37]. Most studies (11) were published in the last five years, from 2016 to 2021, followed by nine studies from 2010 to 2016, and 4 studies from the year 2000 to 2009. Most of the studies were conducted in Asia (48%), Europe (24%), North American (20%), and 4% were conducted in Africa and Australia. Nine studies specified the methods used to commit suicide in the elderly, while other studies did not specify the approach adopted in suicide. About 70% of the studies identified mental illness (specifically depression) as the reason for suicide in the geriatric population. Twenty-three studies (96%) defined elderly starting from 60 or 65 years and above. Table 1 reports on the prevalence of depression in the elderly population, whilst Table 2 reports on the correlates of thoughts of death, suicidal behavior, and death by suicide in the elderly. Table 3 summarizes studies that report on the methods used by the elderly who die by suicide.

## 4. Discussion

This review highlights the high prevalence of major depressive disorder in the elderly and other correlates [38,39,40]. This literature review included studies from 2000 to 2021, which reflects the time of modernization where more internet and online activities have been on the rise [41]. In many countries, migration of the younger generation to their respective nuclear families have left the geriatric population burdened and their mental health not adequately met [41]. The majority of the studies on depression and suicide in the elderly population were published between 2016 and 2021, which suggests there is an alarming health concern in this population. Annually, The International Day of Older Persons on 1 October is observed to mark the need to give ear to the elderly well-being and other peculiar needs [42].

Table 1 suggests that the prevalence estimates of major depressive disorder in the elderly ranged from 5.37 [43] to 56% [39]. The review also indicates that depression is a major reason for suicide in the elderly [39,44,45]. Some studies reported a direct correlation between depression and death by suicide [46,47,48,49]. Other studies identified other mental health illnesses as the reason for suicide in the elderly [2,50,51]. (Table 2). Few of the studies did not report relationships between major depressive disorder and death by suicide [5,52,53,54]. Other correlates of death by suicide in the elderly included; physical illnesses [54,55,56], low socioeconomic factors, living alone, and losing a loved one [53,57]. One of the articles reviewed revealed that sexual dysfunction and cuckoldry contributed to depressive symptoms in the elderly, which predisposes them to suicidal ideation and even death by suicide [5]. Six articles used validated scales like GDS and Beck’s inventory scale and the Diagnostic and Statistical Manual of Mental Disorders in diagnosing depression in the elderly [39,43,44,45,48,49]. Nine articles identified the dominant methods used by the elderly to complete suicide [2,5,37,50,56,57,58,59,60].

### 4.1. Demography and Suicide

Gender differences must be taken into account when suicide is mentioned. Generally, suicide attempts are higher in females than in males [61], and the same for the prevalence of anxiety and depression [62]. Females have a 1.5 to 3 higher prevalence of depression compared to males [22]. Elderly women and widowers have high rates of depression as well [28]. However, most countries have suicide rates of 2–3 times higher in males than in females, which could be due to a male preference for higher lethality methods and the reluctance of males to seek help [63].

The male to female suicide ratio in most Western countries is approximately 3:1, while on the contrary, many Asian countries have the reverse of this ratio [64]. A longitudinal study in the USA disclosed that male gender and higher-income projects lethal suicidal ideation [65]. Depressed elderly males are usually likely to report suicidal ideations [66]. Some researchers suggest that the ratio of suicide rate in men compared to women is higher [67]. Women have a high prevalence of suicidal attempts, while completed suicide is successful with men [56]. One significant rationale is the violent methods used by men for suicide. The use of violent methods for suicidal acts increases the likelihood of completed suicide. Gender and age have proven to be analytic risk factors in determining suicide methods [68].

Deaths by suicide are lower among young people, while suicides occur more frequently in the elderly, 65–74 age group [63]. A retrospective study of autopsies and police reports of suicide from 1979 to 2015 realized that, by age, the most prevalent group for suicide was 60 to 69 years until 1990, when there was a paradigm shift for the highest to be 70 to 79 years [2]. According to Crestani et al. (2019), data by gender from the study states that being married does not affect the risk of suicide by men [2].

Liu et al. (2018) researched suicide behaviors among the elderly and non-elderly. In the study, no significant differences in education level, religion, family history of suicide, nor pesticide storage in the household influence completed suicide [1], however, it somewhat differs in several sociodemographic and psychological factors. Elderly living without a spouse, poor family economic status, and poor social support increased exposure to suicide [69,70].

While other studies have not correlate educational level and depression or suicide, a few studies have. A study in China suggested that lower educational status, poor living conditions, and reduced social interaction with family members poses a risk for lifetime major depressive disorder [71]. Interestingly, phone calls and visits of family members were found to be linked to major depressive disorders [71].

### 4.2. Correlates of Death by Suicide in the Elderly

A retrospective review of autopsy reports of individuals aged 60 and older who died by suicide in Turkey between 2005 and 2014 indicated that the majority of the suicides happened in the summer and specifically in June and July [58].

Psychiatric and physical illnesses [2,51,55,56], functional impairment [35,58] and social/economic factor [50,59] may contribute to suicide in the elderly. Substance abuse and hospitalization for any physical health problems and diagnoses related to injuries also predict suicide [65,72].

### 4.3. Mental Illness

Studies have shown that mental health problems strongly correlate with suicide in the elderly [2,50]. More than half of the studies (autopsies) showed that death by suicide among the elderly is preceded by some psychiatric disorder [46,51]. In the general population, mental disorders are perceived as increasing vulnerability to suicidal behavior, attempted and completed suicides [73]. However, many researchers show psychiatric conditions, particularly depression, as a factor that leads to elderly suicidal behavior [9] since the predominant mental health problem among the elderly is depression [74]. Other studies estimated that 95% of the elderly people who die by suicide had been diagnosed with some mental disorder prior to their death [48,55,75]. Due to these observations, regulatory agencies and professional organizations recommend that physicians routinely screen for depressive symptoms [76]. Other psychiatric comorbidities may contribute to suicide among the elderly. Schizophrenia, dementia, anxiety disorders, and personality disorders have proven to increase risk of suicide in elderly suicides, but to a lesser degree [60,77]. Perceived stress has also proven to be a vital direction that increases the risk of suicide among the elderly population diagnosed with major depressive disorder [78].

### 4.4. Major Depressive Disorder

The diagnostic criteria for major depression in the DSM-5 includes either sadness or anhedonia, with a total of five or more symptoms over two weeks [22]. One important distinction between the DSM-5 and the DSM-IV is that it defined major depressive disorder with the inclusion of bereavement in the former and not in the latter [22,79]. When the elderly are depressed, thinking may be impaired, making it difficult for a logical verdict [80]. In various studies, the definition of late-life depression is used for major depressive disorder, which occurs at age 60 and above for the first time [81,82]. These studies indicate that depression occurring in the elderly for the first time differs from the depression occurring at earlier ages. In terms of clinical presentation, prognosis, and response to treatment [83]. The literature shows that about 5% of major depressive disorder occurs in community-dwelling older adults, and 8 to 16% of older adults have clinically significant depressive symptoms [84]. This result is in agreement with previously reported findings of the elderly experiencing major depressive disorders [39,43]. During a psychological autopsy study of completed suicide in later life, sixteen out of eighteen subjects were diagnosed with major depressive disorder [85], which contributed to their death. Furthermore, a postmortem from psychological autopsy to ascertain the reason behind the suicide act of the elderly proved that 71 to 95% were diagnosed with the mental health disorder before suicide [55,86,87]. A Case-control study in four countries and one large urban area in central England established that 63% of 100 sample size of elders who died by suicide, suffered depression before the act [46].

Studies have shown that rates of major depressive disorder increase with medical morbidity. Five studies suggested psychiatric comorbidities correlated with suicide in the elderly population [2,50,51] with a high rate of 37% after acute care hospitalizations [29]. Major depressive disorder has been shown to be the commonest psychiatric diagnosis in elderly suicide victims, and it is also the main cause of suicide in the geriatric population [29,88]. Other studies suggested that a feeling of hopelessness is also a common symptomatology in elderly suicide victims [37,39,53,69,70]. Nonetheless, depression usually coexists with other medical health conditions and disabilities and can be provoked by diseases that affect the elderly such as diabetes mellitus, stroke, heart disease, Alzheimer’s disease, Parkinson’s disease, and arthritis [10,70,89,90]. In Western and Asian countries, depression as a risk factor of suicidality among the elderly has been confirmed [5]. Elderly men and women experiencing major depression are likely to have suicidal ideation and are more likely to die by suicide [29]. An epidemiological study in an elderly community also reported mild dysphoria and severe depression among the elderly age group [26]. Paradoxically, mild forms of depressive symptoms reduce the quality of life and increase the sense of hopelessness, resulting in suicide in the elderly and suicidal ideation [91]. A study of 969 people aged 75 years and over revealed that 13.3% of the subjects had suicidal thoughts, notwithstanding, 26.7% had major depressive disorder, while 50% were depressed [92]. The interpretation of these studies is akin; suicidal ideation and completion are correlated to major depressive disorder. The characteristic symptoms of elderly suicides with depression are less described; moreover, it aids in fishing out predictors of suicide [93]. A study by Conwell et al. (1996) reported that 76% of elderly suicide victims had psychopathology; 54% with major depression, and 11% with minor depression [29,94]. There is complexity in the depressed elderly and other predictors in elderly people who have already been diagnosed with depression. Most comprehensive studies on elderly suicide have reported the prevalence of major depression and other mood disorders ranging from 60 to 90% during an exhaustive research [93]. Depression cannot be speculated as the sole predictor of suicidal behavior at the expense of other psychiatric comorbidities [51,95]. Suicide is a complex event with multiple causes [19]. According to the literature, there are various predictors of suicide in old age [35,96]. Before the elderly die by suicide, approximately 30% of this population express their desire to die to a close contact [97]. It is quite unfortunate family/close contacts do not respond to this pressing need; hence professional assistance is not sought. Report has shown that approximately six million Americans 65 years or older are affected by depression, but only 10% of those affected receive treatment [90,98].

### 4.5. Physical Illness

The existence of serious illnesses is another precursor and a significant risk factor for suicide among the aged, especially cardiovascular conditions, with prevalence ranging from 34 to 94% [55,99]. Cancer and liver conditions may cause depression in the elderly, while medications to treat pain may be abused by some elderly [57]. Malignancies accompanied by severe pain in the elderly also have a high association with suicide [57]. Studies suggest that many elderly die by suicide because they feared they had cancer, rather than actually dying by suicide due to cancer [100]. A study by Brown et al. (1986) revealed that, among 44 terminally ill elderly patients, one out of four expressed a desire to die by suicide [101]. In a controlled psychological autopsy study of suicide in late life, eight victims (all men) believing they had cancer played a major role in the decision to end their lives [102]. Most of these numbers had diagnosable major affective disorders, but none had sought mental health care [102]. Although physical illness and functional impairment increase the risk of suicide in the elderly, their influence appears to be mediated by depression [47].

Whereas physical illness correlates directly to suicide in the elderly [54], Shin et al. (2020) also revealed that there is an independent relationship between physical health status and suicide behavior in the case of elders [60].

### 4.6. Social/Economic Factor

Sociopsychological autopsies among 122,044 dead elderly subjects reported that social factors promoted suicide in these elderly [85,103], contributing to depressive symptoms [5,69]. These include financial problems, poor occupation, relationship difficulties, family conflicts, social isolation and loneliness [5,19,59]. Psychosocial stressors such as a spouse or loved one’s death and traumatic mourning [9] may trigger a depressive episode, although transient reactions to major losses can resemble depression [5,9,70,84]. In addition, the same researchers found that limited social interaction contributes to suicidal ideation and suicide in the geriatric population [104], making inadequate social interaction a major problem in the elderly [19]. A sense of interaction and socialization will minimize suicide in there elderly and is a protective factor, even when the elderly are not faced with mental disorders [103]. Good social support, therefore, serves as a protective factor against suicide in the elderly [1]. Some authors distinguish between living alone and loneliness [53]. Interviews with the next of kin of the deceased elderly revealed that most elderly lived alone before their death by suicide [53]. Living alone can be the decision of the elderly; on the contrary, loneliness may also occur despite living with people [19]. In a retirement community, hopelessness was found to be a predictor of suicide [105].

Somatic symptoms disorder is another risk factor for suicidal thoughts for older adults in the community [106], which may not be associated with depressed older adults [66].

### 4.7. Substance Use

The rate of substance use disorders among older adults who die by suicide is minimal, with a prevalence range of 5 to 40% [65,72]. However, these values should not be underrated. The toxicological screening was done at the time of death for 96 Honolulu older adults, and analysis revealed about 54% alcohol or possible habituating substance misuse on postmortem [107]. Although women are twice as likely as men to meet the criteria for major depression, they are one-fourth less likely than men to commit suicide [24]. Men who are depressed have a higher prevalence of comorbid alcohol and substance abuse than women [24,108].

Studies have observed that depression in elderly suicide is usually without comorbid substance abuse or personality disorders compared to young adults [109]. Substance use without any comorbid affective disorder is unlikely among the elderly [94,109]. A study to compare substance abuse without comorbid mood disorder in two age groups revealed 39% of the 16–30 age group and 8% in the 60–88 age group experience substance abuse without comorbid mood disorder [99]. Only substance abuse or other mental health problems in late life suggest that dual analysis may be a less essential risk component for elderly suicide than young adults [99]. An increase in alcohol consumption may awaken depressive symptoms and anxiety. Moreover, this may diminish social support and trigger suicide. Older men who continue to abuse alcohol in conjunction with stress from society may be fatal [109]. Alcoholism and late social support can be a trigger for completed suicide [109].

### 4.8. Methods of Suicide

A summary of some of the commonest methods used in suicide is seen in Table 3. Studies have shown that preferred suicide methods differ by age and country [58]; irrespective of country-specific suicide patterns, hanging, pesticide suicide, and firearm dominate [110]. All articles which researched the methods of suicide in this review reported hanging as the dominant method [2,37,60]. However, Altınöz et al. (2019) found that firearms are commonly used in men while jumping from a height was more common among women [50]. It is estimated in the USA that over 60% of the elderly who completed suicides use firearms, with white men engaging in this practice habitually [93].

A systematic review conducted in China revealed that pesticides are frequently used for suicide in rural areas among the general population [111]. Ingestion of agricultural chemicals or rat poison was the most common method (34.3–66.6%) [111]. Other methods include drowning in rivers or wells, jumping from heights, poisoning with other substances, traffic accidents, electrocution, and carbon monoxide poisoning [112,113]. In India, drowning, hanging, and poisoning are ranked the common methods of suicide (63%) [112,114]. This finding contradicts Western countries, where firearms, hanging, and drug ingestion are adopted in suicide, with the former being the commonest [115]. Despite the significance of depressive disorder in elderly suicide, most studies report considerably insufficient/inept use of antidepressants before death [57,116]. A psychiatric assessment among 69 old adults in southern Sydney revealed 81% of the elderly 65 years and over attempted suicide with overdoses of medications like narcotic pain killer and diuretic use; benzodiazepine represents 64% of this group [57,110]. Violent methods of suicide are less common with aging [100]. Methods such as firearm suicide and hanging are more frequent among men, while women choose poisoning or drowning, which are less violent and less lethal [110]. A study of 538 subjects aged 60 years on over showed that 32.52% hanged themselves as means of dying by suicide. Out of that number, 27.88% were men while 4.64% were females [2]. The same study revealed that fall from height was the second most common method used after the former, then drowning and firearm [2]. Kim et al. (2016) researched the suicide attempts in adults and the elderly population in Korea and emphasized that poisoning with pesticides and caustics was more common in the elderly while cutting/piercing was more common in the non-elderly. This concludes that most elderly suicide attempters prefer less-lethal methods [117]. Most of the older people stay alone, making elderly suicide occur at home. They are usually fragile and may be experiencing some physical impairments, making them unable to leave their homes where they choose to die by suicide [58].

### 4.9. Public Policy and Practice Implications

Improved detection and early interventions are crucial in preventing suicidal attempts and completed suicides. Depression which is a vital predictor of suicide must be targeted and treated. Interpersonal psychotherapy and cognitive–behavioral therapy can be effective, yet appointments are required [118]. Notwithstanding, internet-delivered cognitive behavioral therapy may be recommended [98]. Supportive text messages programs like “Text4Hope” have proven to reduce depression and suicidal ideation effectively [119] and can be used to promote social connectedness and healthy aging. The National Strategy for Suicide Prevention emphasized that detection and treatment of depression is an approach to prevent late-life suicide [120]. Policy-makers and mental health professionals need to make a conscious effort to improve mental health services among the geriatric population, reduce the risk of suicide and improve the quality of elderly life. Social interventions such as providing enjoyable activities and increasing interaction with peers should be encouraged. Although this can be difficult to accomplish due to the elderly’s limited mobility and reluctance to participate, they need to be encouraged. The best treatment regimen should be provided for the elderly to treat psychological and physical illnesses to minimize suicide. Nonetheless, most patients with depression and other common mental disorders are treated in general medical settings. Evidence shows that treating depression in patients with physical illnesses positively affects psychological and physical health [121].

### 4.10. Limitations

The search strategy appraised only studies published in the English language. Although much effort was made to identify all relevant studies for this literature review, some relevant studies may be missing, especially those published in other languages. Notwithstanding the limitations of the study, this review provides insight into the prevalence of depression in the elderly, suicidality among the elderly, and some intervention to curtail suicide in the geriatric population.

## 5. Conclusions and Future Directions

The findings from this study provide an understanding of suicide among the elderly. Factors contributing to suicide in the elderly include physical, psychological, and social problems. This review has highlighted the predisposing factors for death by suicide, such as malignancy, medical comorbidities, mental health illnesses including major depressive disorder, and loneliness. Depression was found to be the most dominating predictor of death by suicide among the elderly in most of the studies. Factors such as loss of a loved one, chronic pain, loneliness, financial constraints, and lack of interaction could trigger depression and suicidal thinking [5,53,70]. Older adults (60 years and above) who experience depression or other physical comorbidities are usually faced with difficulties and may have a feeling of hopelessness [53]. Geriatric depression is a complex disorder with multiple risk factors. There is a correlation between age, depression, suicidal ideation, and death by suicide. Any form of action taken by the elderly should be investigated and intervened.

Other mental illnesses also contribute to suicide in the elderly but have a lower effect [47,69]. Physical illness and functional impairment may increase the risk of suicide but are usually triggered by depression. Lack of social interaction is an independently associated risk factor for suicide in later life [47,69]. The study also revealed that gender differences influence the method used in suicide. In the general population, men prefer violent methods of dying by suicide and elderly men often use firearm. Elderly women are often more depressed than elderly men, however, while elderly women have more suicidal ideation, completed suicide is observed more in elderly men. However, Suicide rates are higher in later life than in any other age group [50,89]. Government and policymakers can improve the mental health of the elderly to reduce suicidal risk. E-mental health services can be incorporated to improve geriatric mental health. Early detection of physical illnesses and effective social interaction are recommended to improve the mental health of the geriatric population. There is a need for future studies to evaluate interventions that have been implemented to mitigate social isolation and psychological distress in the elderly population.

## Figures and Tables

**Table 1:** Prevalence of major depressive disorder among the elderly.

Author and Year	Country	Sample Size	Scale Used	Prevalence of MDD
Park J.H. et al. (2010)	Korea	1118	Geriatric Depression Scale (GDS)-Korea	5.37%
Wongpakaran N. et al. (2012)	Thailand	113	Geriatric Depression Scale	23.5%
Booniam S. et al. (2020)	Thailand	803	DSM-IV and Thai Geriatric Depression Scale	18.64%
Padayachey U. et al. (2017)	South Africa	300	Geriatric Depression Scale	40%
Shoib S. et al. (2020)	India	200	Beck’s Depression Inventory	56%
Wærn M. et al. (2002)	Scandinavia	100	DSM-IV	38%

**Table 2:** Correlates of thoughts of death, suicidal behavior, and death by suicide in the elderly.

Name of Author and Year	Country of Origin	Study Design	Targeted Group	Sample Size	Reason for Suicide	Findings
Crestani C. et al. (2019)	Italy	A retrospective study (autopsy/police report)	60 years and above	538	Mental illness	The highest risk of suicide is observed in the age between 70 and 79 years. Pathological factors were revealed in 427 cases. Mental illness was related significantly to suicidal risk.
Bogers I.C.H.M. et al. (2013)	Netherlands	A prospective study	60 years and above	378	Depression and physical injury	Patients reporting thoughts of death but no suicidal ideation were older and more severely depressed, whereas patients with suicidal ideation were also more severely depressed but not older.
Rurup M.L. et al. (2011)	Netherlands	A longitudinal cohort study	58–98 years	1794	Psychiatric comorbidity; dysthymia and panic attack.	Of those who wished to die, 67% had depressive symptoms, and 20% suffered from a depressive disorder.
Altınöz A.E. et al. (2019)	Turkey	A retrospective study	65 years and above	978	Mental illness and financial difficulties.	The most common cause of suicide was financial difficulty for men and marital conflict for women. The most frequent suicide method among older adults of both sexes was hanging. In all age groups, firearms use was more common among men and jumping from a high place was more common among women.
Conejero I. et al. (2018)	Korea	A prospective longitudinal study	60 years and above	1548	Physical illness	Independent relationship between physical health status and suicidal behavior in the elderly. High correlation with the history of suicide attempts in the elderly, and there is independence between depression factors and suicide.
Bogers I.C. et al. (2013)	Netherlands	A prospective multi-site naturalistic study	60 years and above	378	Depression	In depressed older persons, thoughts of death and suicide differ in relevant demographic, social, and clinical characteristics, suggesting that the risks and consequences of the two conditions differ.
Bickford D. et al. (2020)	San Francisco	A retrospective study	65 years and above	225	Perceived stress	Perceived stress was found to be a risk for suicidal activity in depressed older adults.
Booniam S. et al. (2020)	Thailand	A retrospective study	mean age of 69 years	803	Major Depressive Disorder (MDD)	MDD was the main predictor for suicidal ideation; however, agoraphobia and poor perceived social support increases suicide risk.
Razai D. et al. (2020)	Iran	A cross-sectional descriptive-analytic study	65 years and above	1601	Physical and mental illnesses	Successful suicides have been rising, from 3.7 in 2008 to 4.37 per 100,000 people in 2014.
Rossom B.C. et al. (2019)	US	Cross-sectional cohort study	65 years and above	203,668	Depression	Depression severity was by far the strongest predictor of suicidal ideation in older adult patients. Older patients with suicidal ideation should be screened for depression.
Shin K.M. et al. (2013)	Korea	A prospective study	60 years and above	1548	Anxiety, depression and stroke	This study suggests that there is an independent relationship between physical health status and suicide behavior in the case of elders.
Shoib S. et al. (2020)	India	A cross-sectional study	65 years and above	200	Depression	Depression had a positive correlation with suicidal ideation. Hopelessness and suicidal intent had a more significant positive correlation.
Bergman L.T. et al. (2011)	Israel	A retrospective study	65 years and above	78	Physical illness	Suicidal patients scored higher in the vascular and respiratory section of the cumulative illness rating scale; higher rates of illness among suicidal elderly patients.
Liu B.P. et al. (2018)	China	A paired case-controlled	60 years and above	190	Not living with a spouse, depressive symptoms	The influence of negative life events increases the risk of suicide in the elderly.
Sun W. et al. (2010)	China	A cohort Study	65 years and above	56,088	Depression and physical illness	Depressive symptoms were associated with all-cause mortality in men and with suicide in both sexes.
Kaya A. et al. (2020)	Turkey	A retrospective study(autopsy)	60 years and above	17,942	Asphyxia, CNS injury and physical illness	In the summer, June and July, the suicides occurred more frequently in the 65–74 age subgroup.
Adinkrah M. et al. (2020)	Ghana	A descriptive study	60 years to 65 years	40	Lack of finance, indebtedness, cuckoldry, sexual dysfunction, grief or marital breakdown.	Elderly persons who died by suicide were male, aged 60 to 65 years old, and of low income.
De Leo D. et al. (2013)	Australia	A case-control study	60 years and above	261	Psychiatric diagnosis, hopelessness, past suicidal attempts and living alone	Older adult suicides showed a significantly lower prevalence of psychiatric diagnoses (62%) when compared to middle-aged suicide cases (80%). In both age groups, subjects who died by suicide were significantly more likely to present a psychiatric diagnosis.
Chan H.L. et al. (2011)	Taiwan	A Cross-sectional Study	65 years and above	3853	Physical illness and depressive symptoms	The point prevalence of elderly suicidal ideation was 6.1%. Female gender, age over 85 years, low level of education, single status, unemployment, no income, disability, current smoking, self-perceived bad to very bad health, depressive symptoms, various physical disorders, and pain symptoms were strongly associated with suicide ideation.
Conwell Y. et al. (2002)	Monroe (US)	A case-control study (psychological autopsies)	60 years and above	238	Depressive illness	Completed suicides had a more depressive illness, physical illness burden and functional limitations. They were more likely to be prescribed antidepressants, anxiolytic agents and narcotic analgesics. Among depressed subjects, affective symptom severity and emotional dysfunction distinguished suicide completers.
Wærn M. et al. (2002)	Scandinavia	A case-control study (psychological autopsies)	65 years and above	100	Depressive disorder	Ninety-seven percent of the suicide victims fulfilled the criteria for at least one DSM-IV axis I diagnosis, compared with 18% of the living comparison subjects. Recurrent major depressive disorder was a very strong risk factor for suicide, as was substance use disorder.
Turvey C.L. et al. (2002)	US	A longitudinal cohort study	66 years and above	14,456	Depressive symptoms, perceived health status, sleep quality, and absence of loved one	This study provided additional information about the context of late-life depression that also contributes to suicidal behavior: poor perceived health, poor sleep quality, and limited presence of a relative or friend to confide in.
Wongpakaran N. et al. (2012)	Thailand	A cross-sectional descriptive study	63 years- 94years	113	Major Depressive Disorder	23.5% met the criteria for current major depressive episodes and suicide risk was reported for one-third of the elderly.
Harwood D. et al. (2001)	UK	A descriptive and case-control study	60 years and above	154	Depression, personality disorder, and personality trait	77% of the suicide sample had a psychiatric disorder at the time of death, most often depression (63%).
Voaklander D.C. et al. (2008)	British Columbia	A case-control studies	66 years and above	602	Lower socioeconomic status, depression and physical illness.	The annual rate of suicide is 13.2 per 100,000. Firearms were the most common mechanism (28%), followed by hanging/suffocation (25%), self-poisoning (21%), and jumping from height (7%). There was an elevated risk for those prescribed inappropriate benzodiazepines and those using strong narcotic pain killer.

**Table 3:** Summary of the most common methods used in suicide in the elderly.

Author and Year	Methods of Suicide
Crestani C. et al. (2019)	Hanging Fall from height Firearm
Altınöz A.E. et al. (2019)	Hanging Firearm Jump from height
Razai D. et al. (2020)	Hanging Poisoning
Kim K.H. et al. (2016)	Drug overdoses Pesticide or caustics
Rossom B.C. et al. (2019)	Hanging
Shin K.M. et al. (2013)	Hanging
Adinkrah M. et al. (2020)	Hanging and firearm
Kaya A. et al. (2020)	Hanging
Voaklander D.C. et al. (2008)	Medication overdose (benzodiazepine) Firearm

## Data Availability

This review is not associated with any primary or secondary data.
